# Impact of Staging, Histologic Grading, and Racial Background on Lip Cancer Survival in the United States: Insights from the SEER Database (2010–2020)

**DOI:** 10.1158/2767-9764.CRC-25-0075

**Published:** 2025-09-02

**Authors:** Muhammad Taqi, Syed Jaffar Abbas Zaidi

**Affiliations:** 1 Community Dentistry Department, Dow University of Health Sciences, Karachi, Pakistan.; 2 Oral Biology Department, Dow University of Health Sciences, Karachi, Pakistan.

## Abstract

Lip cancer has a distinct biological behavior within head and neck oncology, although population-based survival studies remain scarce. We analyzed data from the United States Surveillance, Epidemiology, and End Results (SEER) program to assess the prognostic value of tumor stage, histologic grade, and race in lip cancer diagnosed during 2010 to 2020. A retrospective cohort of 6,717 invasive lip tumors was extracted from 17 SEER registries. Cause-specific survival was estimated using Kaplan–Meier curves and compared with log-rank tests. Cox proportional hazards regression generated HRs with 95% confidence intervals. Among 4,273 lip cancer cases with SEER staging, survival varied significantly by disease extent. Localized tumors had a 94% lower risk of cancer-specific death compared with distant/regional metastasis, with median survival times of 97 and 18 months, respectively. Histologic grade correlated with survival in univariate but not multivariate analysis (after adjusting for stage and race). White and Asian/Pacific Islander patients had better survival, whereas American Indian/Alaska Native patients (0.4% of the cohort) showed a significantly elevated risk, warranting further study in this underrepresented group. In this national cohort, disease stage at diagnosis and race were key survival determinants in lip cancer. Localized tumors reduced mortality by 94% versus distant disease. Histologic grade added no further prognostic value after accounting for stage and race, underscoring the need for longer follow-up and better registry data.

**Significance:**

This SEER-based study provides the first lip-specific survival curves, revealing localized disease reduces mortality by 94% versus distant metastasis. Race (American Indian/Alaska Native patients faced triple the mortality risk) outweighed histologic grade in prognosis. Limited tumor–node–metastasis data highlighted registry gaps, whereas nodal sampling trends supported early regional assessment. Findings refine risk stratification, expose disparities needing targeted interventions, and set benchmarks for future research in this uncommon but clinically significant cancer.

## Introduction

Lip cancer, a subset of oral cancers, represents a unique clinical entity with distinct epidemiologic characteristics and treatment challenges. Although less common than oral cancers of the tongue or oropharynx, lip cancer raises significant concerns because of potential disfigurement and its impact on the quality of life (1). Prognosis is influenced by tumor stage, histologic grade, and demographic characteristics, although survival data across various population groups remain limited (2, 3).

The tumor–node–metastasis (TNM) staging system is widely recognized for classifying cancer spread, guiding both treatment and prognostication (4, 5). The Surveillance, Epidemiology, and End Results (SEER) database offers a valuable resource for large-scale epidemiologic studies by providing detailed information on incidence, staging, and survival (6, 7). By analyzing lip cancer cases diagnosed between 2010 and 2020, the present study captured recent trends in patient demographics, tumor characteristics, and management (6). These years encompass advancements in surgery, radiotherapy, and systemic treatments that potentially influence survival outcomes (8, 9). They also reflect evolving demographic changes in the US population, highlighting shifts in cancer incidence and mortality (10). This study evaluated the state of lip cancer treatment during this period and identified potential areas for improvement.

Lip and oral cancers are both categorized as head and neck malignancies but differ considerably in their clinical and epidemiologic profiles. Lip cancer is typically diagnosed at early stages, with an estimated 75% of cases being localized, leading to a 5-year survival rate of approximately 90% (11, 12). This favorable prognosis often reflects a predominance of basal cell carcinoma, which grows slowly and rarely metastasizes (13). In contrast, oral cancer, predominantly squamous cell carcinoma (SCC), is often diagnosed at advanced stages, with only 30% to 40% of cases detected early, leading to a lower 5-year survival rate of approximately 65% (14, 15).

Demographic factors further distinguish between these cancers. Lip cancer primarily affects older Caucasian men with high UV exposure, whereas oral cancer is more prevalent among non-Caucasians, including Hispanics, Asians, and African Americans (16). Risk factors vary; sun exposure predominates for lip cancer, whereas tobacco, alcohol, and human papillomavirus play larger roles in oral cancer (17). Tailored prevention and detection strategies must consider these differing risk profiles.

Despite the clinical importance of lip cancer, research in this specific area remains relatively sparse, as many studies have grouped lip cancer with other oral malignancies (18). Consequently, the unique demographic, pathologic, and therapeutic factors that influence lip cancer outcomes are not well understood. This gap limits clinicians’ ability to develop targeted interventions to improve the prognosis of patients with lip cancer.

Research challenges include obtaining data from a large patient population. In this study, we used the SEER cancer database maintained by the American Cancer Society and American College of Surgeons. The SEER is the gold standard for cancer registries in the Unites States and worldwide. To maintain quality, regional registries must sign contractual agreements and meet SEER standards before transmission (19). The United States uses this database to determine the incidence, mortality, and morbidity rates of malignant tumors in several states and counties. The tumor information in the database was standardized and unified using SEER* Stat software, which is regularly updated and released. Using this application, clinical researchers can easily access the data, making it an excellent source of clinical data. Moreover, the SEER database covers approximately 34.6% of the US population. Research based on the SEER database offers high clinical value owing to its strong statistical efficiency (15).

Existing literature on oral cancers primarily concentrates on more common forms, such as SCC of the tongue and oropharynx, often neglecting lip cancer as a distinct entity. Consequently, there is a shortage of data specific to the characteristics and survival outcomes of patients with lip cancer, especially in relation to demographic factors, tumor staging, and histologic grading. However, there is a lack of detailed lip cancer-specific analyses, especially regarding survival outcomes by stage and grade.

Accordingly, this study aimed to characterize the demographic, pathological, and treatment-related features of US lip cancer cases diagnosed between 2010 and 2020 and to test the independent effects of tumor staging, histologic grading, and race on overall survival. We first used the *χ*^2^ test to compare survivors and nonsurvivors. We then used Kaplan–Meier curves and multivariable Cox models to quantify how stage, grade, and race (with age included as a covariate) shaped the survival trajectories. By interrogating these predictors within a lip-only cohort, this study fills a key gap in the literature by providing evidence that can guide clinicians, inform future research, and support interventions aimed at improving outcomes for this specific tumor site.

## Materials and Methods

### Study design

A retrospective cohort design was used, using data from the SEER database. This database compiles population-based cancer incidence and survival data from the US registries. This study encompassed malignant lip cancer cases diagnosed between 2010 and 2020, capturing changes in incidence, staging, and treatment over the past decade. The selection of the 2010 to 2020 period was deliberate, aiming to capture the most contemporary practices in the diagnosis, staging, and treatment of lip cancer, reflecting advancements in medical techniques and shifts in demographic patterns.

### Data collection

Data were obtained from 17 SEER registries (20), yielding 6,717 malignant lip cancer cases. Patients ranged in age and were classified into four categories: <50 years, 50 to 64 years, 65 to 70 years, and >75 years, balancing both statistical stability and clinical interpretability.

The histologic type codes for cancer included in our study were 8430, 8502, 8010, 8200, 8550, 8525, 8072, 8560, 8070, 8071, 8140, 8147, 8562, 8083, 8720, 8052, 8123, 8075, 8000, 8074, 8082, 8032, 8772, 8982, 8246, 8033, 8076, 8310, 8440, 8084, 8073, 8746, 8022, 8094, 8255, 8050, 8450, 8500, 8091, 8090, 8092, 8095, 8413, 8407, 8004, 8401, 8410, 8390, 8745, 8098, 8081, 8102, 8480, and 8542, in accordance with the third edition of the International Classification of Diseases for Oncology.

The histologic type codes used for cancer classification were in accordance with the third edition of the International Classification of Diseases for Oncology, with specific codes for lip cancer (C000–C006 and C008–C009). Variables included age, gender, race, marital status, tumor location, chemotherapy, radiation, systemic therapy, surgery details, tumor grade, and survival status. Because SEER data are publicly available and deidentified, no Institutional Review Board approval or ethical consent was required.

### Sample size

This study used a nonprobability sampling method, specifically a convenience sampling technique, because the dataset comprised all eligible cases recorded within the specified timeframe and regions. The extensive population coverage of the SEER database enhanced the generalizability and reliability of the findings. For this study, all malignant cases of lip cancer diagnosed between 2010 and 2020 in the age groups (5–9 years to 85+ years) were included, ensuring a comprehensive dataset that provides robust statistical power for detailed subgroup analyses. This broad inclusion enhances representativeness and statistical power.

### Statistical analysis

Data were analyzed using Statistical Package for the Social Sciences (SPSS) version 21. The demographic characteristics of lip cancer patients were compared between survivors and nonsurvivors using the *χ*^2^ test to evaluate statistical significance. The treatment and pathologic characteristics were similarly assessed using *χ*^2^ tests to explore their association with survival status.

Time-to-event outcomes were analyzed using Kaplan–Meier survival curves, and the log-rank test was used to compare survival distributions across groups. Cox proportional hazard regression was used to estimate HRs and 95% confidence intervals (CI) for predictors of survival, including staging, histologic grade, and race. The survival analysis presented in this study was based on cancer-specific survival using the SEER Cause-Specific Death Classification variable, as derived from the SEER database, in which death from lip cancer is considered an event of interest. This variable was selected because it accounts for site-specific mortality and is curated by the SEER to minimize cause-of-death misclassification. For survival analyses, cases lacking data were excluded.

The American Joint Committee on Cancer TNM staging system is not routinely available in SEER data for all diagnosis years. To address this limitation and ensure a comprehensive and consistent staging variable across the entire study period, we extracted the Summary Stage of 2000 (1998–2017) from the SEER database. This variable categorizes cancer extent into standardized groups: *in situ*, *localized*, *regional*, *distant*, and *unknown*. It is available for all cancer sites and years and thus provides a reliable measure of disease extent at diagnosis for population-based analyses. For additional verification and refinement, we also derived an approximate stage grouping based on the available TNM components (T, N, and M) wherever complete TNM information was present. This derived staging was used to cross-validate the Summary Stage categorization and ensure consistency in classification.

Cases with unknown staging were excluded from stage-stratified survival analyses. This dual approach, which involved using the SEER Summary Stage and derived TNM-based staging, allowed us to mitigate the effects of systematic missingness and maximize the use of available data across all years in the dataset. Statistical significance was defined as a two-tailed *P* value of < 0.05.

### Data availability

All data used in this study are publicly available from the SEER program (https://seer.cancer.gov) of the NCI. Processed data generated during this study are available from the corresponding author upon reasonable request.

## Results

Table 1 presents the demographic characteristics of patients with lip cancer stratified by their vital status (alive vs. deceased). Race was significantly associated with survival (*P* = 0.001). White patients comprised the largest proportion of the cohort, with 70.8% alive and 29.2% deceased patients. Asian or Pacific Islander patients showed a similar survival profile, with 72.2% survival. However, Black patients had a slightly higher mortality rate (35.1% deceased), and American Indian/Alaska Native (AI/AN) patients exhibited the highest mortality rate (44% deceased). Notably, among patients of unknown race, an overwhelming majority (97.7%) were alive, which may reflect data classification artifacts.

**Table 1:** Demographic characteristics of patients with lip cancer stratified by survival status (row percentages shown). *P* values from *χ*^2^ tests

Variable	Alive	Deceased	*P* value
Race	​	​	​
White	4,393 (70.8)	1,812(29.2)	0.001
Black	48 (64.9)	26 (35.1)	
Asian or pacific islander	83 (72.2)	32 (27.8)	
AI/AN	14 (56)	11 (44)	
Unknown	291 (97.7)	7 (2.3)	
Gender	​	​	​
Male	3,420 (71.2)	1,386 (28.8)	0.018
Female	1,409 (73.7)	502 (26.3)	
Marital status	​	​	​
Married	2,164 (73.6)	776 (26.4)	0.001
Single	580 (72)	225 (28)	
Widowed	304 (47.1)	342 (52.9)	
Divorced	317 (70.9)	130 (29.1)	
Separated	30 (62.5)	18 (37.5)	
Unmarried	16 (66.7)	8 (33.3)	
Unknown	1,418 (78.5)	389 (21.5)	
Age in years	​	​	​
<50	588 (91.9)	52 (8.1)	0.001
50–64	1,740 (84.9)	310 (15.1)	
65–74	1,323 (76.6)	404 (23.4)	
≥75	1,178 (51.2)	1,122 (48.8)	

*χ* ^2^ test.

Gender differences were also statistically significant (*P* = 0.018), with female patients demonstrating a slightly better survival rate (73.7% alive vs. 71.2% for males).

Marital status revealed substantial differences in the survival outcomes (*P* = 0.001). Married individuals had the highest survival rate (73.6%), whereas widowed patients exhibited the poorest outcomes, with more than half (52.9%) deceased. Single, divorced, and separated patients had intermediate mortality rates. Interestingly, patients with unknown marital status had relatively favorable survival rates (78.5% alive), possibly reflecting reporting or classification discrepancies.

Marital status was significantly associated with survival outcomes (*P* = 0.001). A higher percentage of married individuals were alive (73.6%) compared with those who died (28.8%). The proportions of single, divorced, separated, and unmarried individuals were comparable between groups.

Finally, the age distribution was strongly associated with survival (*P* = 0.001). The survival rates declined sharply with increasing age. Among patients less than 50 years old, 91.9% were alive, compared with 84.9% for those ages 50 to 64 years, 76.6% for those ages 65 to 74 years, and only 51.2% for those ages 75 years and above. The oldest age group exhibited nearly equal proportions of survivors and nonsurvivors.

**Figure 1:**
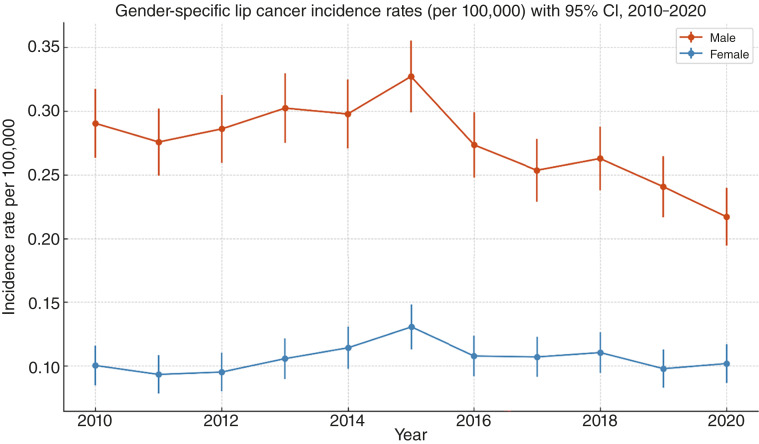
Gender-specific incidence rates of lip cancer per 100,000 population in United States (2010–2020).

Figure 1 illustrates the annual gender-specific incidence rates of lip cancer per 100,000 population size in Pakistan from 2010 to 2020, along with 95% CIs. The male incidence rates were consistently higher than the female rates throughout the study period. The highest male incidence was observed in 2015 at approximately 0.33 per 100,000 (95% CI, 0.30–0.36), declining to 0.22 (95% CI, 0.19–0.24) by 2020. The female incidence remained relatively stable, peaking in 2015 at 0.13 per 100,000 (95% CI, 0.11–0.14) and declining to 0.10 (95% CI, 0.09–0.12) by 2020. CIs were narrower for males because of the larger number of cases, indicating greater statistical precision. These findings highlight a significant gender disparity in lip cancer burden, with males being disproportionately affected throughout the decade.

Table 2 outlines the pathologic characteristics of patients with lip cancer according to their survival status (alive vs. deceased). T classification (tumor size/extent) did not show a statistically significant association with survival outcomes (*P* = 0.573). Across all T stages, the survival rates were relatively similar, ranging from 62.5% to 76.2%. Patients with T2 tumors showed the highest survival rate (76.2% alive), whereas those with T4b tumors had the lowest survival rate (62.5% alive). A large portion of the dataset (*n* = 5,218) had blank entries for T classification, among which 72.2% were alive.

**Table 2:** Pathologic characteristics of alive and deceased individuals with lip cancer with *P* values (row percentages shown)

Variables	Alive	Deceased	*P* value
T classification	​	​	0.573
T1	450 (71.1)	183 (28.9)	
T2	131 (76.2)	41 (23.8)	
T3	80 (69.6)	35 (30.4)	
TX	376 (69.1)	168 (30.9)	
T4a	19 (70.4)	8 (29.6)	
T4b	5 (62.5)	3 (37.5)	
Blanks	3,768 (72.2)	1,450 (27.8)	
N classification	​	​	​
N0	744 (71.4)	298 (28.6)	0.880
N1	17 (73.9)	6 (26.1)	
N2a	12 (75)	4 (25)	
N2b	12 (80)	3 (20)	
N2c	6 (60)	4 (40)	
N3b	14 (70)	6 (30)	
NX	257 (68.7)	117 (31.3)	
Blanks	3,767 (72.2)	1,450 (27.8)	
M classification	​	​	​
M0	1,057 (70.9)	433 (29)	0.192
M1	5 (50)	5 (50)	
Blanks	3,767 (72.2)	1,450 (27.7)	
Grade	​	​	​
Well-differentiated; grade I	1,143 (70.7)	472 (29.3)	0.001
Moderately differentiated; grade II	886 (62.9)	521 (37)	
Poorly differentiated; grade III	130 (42.6)	175 (57.3)	
Unknown	2,664 (78.8)	716 (21.1)	
Undifferentiated/anaplastic; grade IV	6 (60)	4 (40)	
Staging (SEER Summary Stage)	​	​	​
Localized	2,283 (50.4)	2,251 (49.6)	0.001
Regional	69 (34.2)1	133 (65.8)	​
Distant	29 (18.7)	126 (81.3)	​
Blanks	1,598 (87.5)	228 (12.5)	​

Abbreviation: NX, unknown nodal status.

*χ* ^2^ test.

The N classification (regional lymph node involvement) was also not significantly associated with survival (*P* = 0.880). Survival rates were consistent across most nodal stages, with N2b cases showing the highest survival (80% survival) and N2c cases showing slightly lower survival (60% survival). Patients with unknown nodal status and those with blank values had survival rates similar to those of the overall cohort (68.7% and 72.2%, respectively).

The M classification (presence of distant metastasis) showed a nonsignificant trend toward poorer survival in metastatic cases (*P* = 0.192). Among those with M0 (no distant metastasis), 70.9% survived compared with only 50% among patients with M1 (distant metastasis), indicating worse outcomes with metastasis, although the sample size for M1 was small. The majority of patients had blank values for the M classification, with a survival rate of 72.2%.

In contrast, histologic grade was significantly associated with survival outcomes (*P* < 0.001). Patients with well-differentiated (grade I) tumors had a survival rate of 70.7%, whereas those with moderately differentiated (grade II) tumors showed a reduced survival rate (62.9%). The poorest survival was observed among patients with poorly differentiated (grade III) tumors, of whom only 42.6% survived. Among patients with an unknown tumor grade, the survival rate was high (78.8%), possibly due to reporting or classification limitations. Few patients with undifferentiated/anaplastic (grade IV) tumors had a 60% survival rate.

The distribution of vital status varied significantly across SEER summary staging categories (*P* = 0.001). Among patients diagnosed with localized disease, survival was nearly balanced, with 50.4% alive and 49.6% deceased. In contrast, patients with regional stage disease showed poorer outcomes, with only 34.2% alive and 65.8% deceased.

The worst survival was observed in those with distant metastasis, in which only 18.7% were alive and a striking 81.3% were deceased. Interestingly, among the cases with missing or unclassified staging information (*n* = 1,826), a substantial majority (87.5%) were still alive, and only 12.5% died.

Table 3 presents the distribution of treatment modalities and examination of regional lymph nodes in relation to survival outcomes among patients with lip cancer. The “unknown” categories for lymph node examination and systemic therapy sequence were retained in the table for data transparency but were appropriately excluded from statistical comparisons, as noted in the table footnote.

**Table 3:** Distribution of treatment modalities and regional lymph node examination by outcome

Treatment modality	Alive *n* (%)	Deceased *n* (%)	*P* value
Chemotherapy	​	​	​
Yes	96 (57.4%)	71 (42.5%)	0.001
No	4,733 (72.2%)	1,817 (27.7%)	
Surgery–radiation sequence	​	​	​
No radiation or surgery	4,502 (72.6%)	1,728 (27.7%)	0.028
Radiation after surgery	318 (67.3%)	154 (32.6%)	
Radiation before/both	9 (64.3%)	6 (35.7%)	
Systemic therapy and surgery sequence	​	​	​
None	4,737 (72.1%)	1,828 (27.8%)	0.001
Systemic therapy after surgery	84 (60.4%)	55 (39.5%)	
Other/unknown	8 (66.7%)	4 (33.3%)	​
Regional lymph nodes examined	​	​	​
None	4,333 (72.5%)	1,647 (27.5%)	0.002
1–3 nodes	35 (70.0%)	15 (30.0%)	
4+ nodes	280 (65.5%)	147 (34.4%)	
Sentinel lymph node biopsy	34 (75.6%)	11 (24.4%)	
Biopsy/aspiration NOS	13 (44.8%)	16 (55.2%)	
Unknown	134 (72.0%)	52 (28.0%)	​

*χ* ^2^ test.

“Unknown” categories were included in the table for completeness but were excluded from *χ*^2^ analysis. *P* values reflect comparisons among known categories only.

Abbreviation: NOS, not otherwise specified.

Among patients who received chemotherapy, 57.4% were alive and 42.5% died. In contrast, among those who did not receive chemotherapy, a higher proportion (72.2%) were alive, whereas 27.7% were deceased. This unexpected pattern, in which the death rate is higher among those who received chemotherapy, may reflect underlying factors, such as disease severity, in which patients with more advanced or aggressive diseases are more likely to be treated with chemotherapy, which can influence outcomes regardless of treatment efficacy.

The sequence of surgery and radiation also influenced outcomes. Patients who underwent radiation following surgery had a slightly lower survival rate (67.3%) compared with those who received no radiation or surgery (72.6%). Patients who received radiation before or both before and after surgery demonstrated a survival rate of 64.3% (*P* = 0.028).

Regarding systemic therapy and surgery sequence, those who received systemic therapy after surgery had lower survival (60.4%) compared with those who had neither treatment modality (72.1%; *P* = 0.001).

Regional lymph node examination also showed a significant association with survival (*P* = 0.002). Survival was highest in patients who underwent sentinel lymph node biopsy (75.6%) and those with no lymph node intervention (72.5%). In contrast, those who underwent biopsy or aspiration (not otherwise specified) had the lowest survival rate (44.8%).

For survival analysis, participants with complete data were selected in the case of tumor staging, tumor grade, and race. Table 4 presents the results of both the Kaplan–Meier survival analysis and Cox proportional hazards model for tumor staging, tumor grade, and race in patients with lip cancer.

**Table 4:** Survival analysis and HRs for TNM staging and grading

​	Kaplan–Meier survival analysis			Cox proportional hazards model		
	Survival time in months		*P* value	HR	95% CI	*P* value
	Median	95% CI				
Staging	​					
Localize	N.E	N.E	0.001^a^	0.061	0.44–0.86	0.001
Regional	97.0	N.E		0.489	0.339–0.707	0.001
Distant	18.0	7.54–28.54		Reference range		
Grading	​					
Well-differentiated	N.E	N.E	0.001^a^	0.273	0.67–3.08	0.070
Moderately differentiated	N.E	N.E		0.532	0.13–2.14	0.375
Poorly differentiated	N.E	N.E		1.55	0.38–6.34	0.539
Undifferentiated anaplastic	N.E	N.E		Reference range		
Race	​	​	​	​	​	​
AI/AN	N.E	N.E	0.001^a^	3.208	1.43–7.17	0.005
Asian or Pacific Islander	N.E	N.E		2.452	1.61–3.72	0.001
Black	114.0	N.E		2.703	1.64–4.44	0.001
White	N.E	N.E		Reference range		

Abbreviation: N.E, not estimable.

a Log-rank (Mantel–Cox) test.

### Survival analysis by tumor stages

A total of 4,273 patients with available staging data were analyzed to evaluate the impact of the clinical stage on survival outcomes. Of these, 449 (10.5%) deaths were recorded, whereas 3,824 (89.5%) cases were censored. Most cases were diagnosed at the localized stage (*n* = 3,945), followed by regional (*n* = 275) and distant (*n* = 53) stages. The proportion of censored cases was the highest in the localized stage (92.4%) and lowest in the distant stage (28.3%).

Owing to the high censoring rate, median survival times were not estimable for most categories, except for distant and regional stages. Patients with distant-stage disease had a median survival of 18 months (95% CI, 7.5–28.5), whereas regional-stage patients had a median survival of 97 months. Kaplan–Meier analysis using the log-rank (Mantel–Cox) test demonstrated a statistically significant difference in survival distributions across the staging groups (*χ*^2^ = 814.338, *df* = 2, *P* < 0.001), indicating that survival varied markedly by stage at diagnosis.

To further examine the relationship between clinical stage and the risk of death, a Cox proportional hazards model was fitted. The overall model was statistically significant (omnibus test: *χ*^2^ = 812.472, *df* = 2, *P* < 0.001). Compared with patients with distant-stage disease (reference group), those diagnosed at the localized stage had a significantly lower hazard of death (HR = 0.061; 95% CI, 0.044–0.086, *P* < 0.001), whereas patients with regional-stage disease also a significantly reduced hazard of death (HR = 0.489; 95% CI, 0.339–0.707; *P* < 0.001).

These findings confirm that survival is strongly influenced by the stage at diagnosis, with markedly improved outcomes observed in patients diagnosed at earlier stages of disease.

### Survival analysis by grading

A total of 2,327 cases were analyzed to assess survival outcomes based on tumor grade. Among them, 302 (13.0%) deaths were observed, whereas 2,025 (87%) cases were censored. The distribution of cases by tumor grade revealed that most tumors were well differentiated (grade I: *n* = 1,164), followed by moderately differentiated (grade II: *n* = 966), poorly differentiated (grade III: *n* = 189), and undifferentiated/anaplastic tumors (grade IV: *n* = 8). The percentage of censored cases was the highest in grade I (92.2%) and lowest in grade III (63.5%).

The median survival times could not be estimated because of the high proportion of censored observations in all groups. The Kaplan–Meier survival analysis using the log-rank (Mantel–Cox) test indicated a statistically significant difference in survival distributions across tumor grades (*χ*^2^ = 141.745, *df* = 3, *P* < 0.001), suggesting that tumor grade significantly influences patient survival.

Furthermore, a Cox proportional hazards regression model was used to quantify the risk of mortality associated with tumor grade. The model was statistically significant (omnibus test: *χ*^2^ = 141.443, *df* = 3, *P* < 0.001), indicating that the inclusion of tumor grade as a covariate improves the prediction of survival outcomes. However, none of the individual grade categories showed statistically significant HRs compared with the reference group. Specifically, the HR for grade I was 0.273 (95% CI, 0.067–1.110; *P* = 0.070), for grade II was 0.532 (95% CI, 0.132–2.148; *P* = 0.375), and for grade III was 1.555 (95% CI, 0.381–6.345; *P* = 0.539).

Whereas the overall model indicated a significant effect of tumor grade on survival, the wide CIs and nonsignificant individual HRs may suggest potential variability within subgroups or insufficient event rates, particularly in higher-grade tumors.

### Survival analysis by race

A total of 5,305 patients were included in the analysis to assess the impact of race on survival outcomes. Among these, 529 deaths were observed and 1,412 cases were censored. The majority of the patients were White (*n* = 5,098), followed by Asian or Pacific Islander (*n* = 116), Black (*n* = 64), and AI/AN (*n* = 27). The proportion of censored cases was highest among White individuals (90.5%) and lowest among Black individuals (75.0%).

Due to a high proportion of censored cases, median survival times were not estimable for most racial groups, except for the Black population, which had a median survival of 114 months. Kaplan–Meier analysis using the log-rank (Mantel–Cox) test revealed a statistically significant difference in survival distributions across racial groups (*χ*^2^ = 42.226, *df* = 3, *P* < 0.001), indicating that race significantly affects survival outcomes.

To quantify the relative risk of death, a Cox proportional hazards regression model was performed. The model was statistically significant (omnibus test: *χ*^2^ = 42.133, *df* = 3, *P* < 0.001), confirming that race was a significant predictor of survival. Compared with White patients (reference group), the hazard of death was significantly higher for AI/AN patients (HR = 3.21; 95% CI, 1.43–7.18; *P* = 0.005), Asian or Pacific Islander patients (HR = 2.45; 95% CI, 1.61–3.73; *P* < 0.001), and Black patients (HR = 2.70; 95% CI, 1.64–4.45; *P* < 0.001).

These findings highlight racial disparities in survival outcomes, with non-White patients exhibiting significantly increased risks of mortality compared with their White counterparts.

## Discussion

This nationwide analysis of 6,717 lip cancer cases diagnosed between 2010 and 2020 provides contemporary, lip-specific survival benchmarks and clarifies which clinicopathologic factors retain independent prognostic value. Unlike oral cancers of the tongue or oropharynx, lip cancer is often diagnosed early and is dominated by slow-growing malignancies, such as basal cell carcinoma (20–23). Nevertheless, information specific to demographic predictors of lip cancer, staging, grading, and survival remains sparse. Several important findings differ from patterns reported in earlier work that combined lip lesions with other oral cavity sites and therefore require careful interpretation (21).

Although stage is already recognized as the principal predictor of survival in solid tumors, including lip cancer, several critical knowledge gaps justify our analysis. First, almost all large-scale survival studies have pooled lip lesions with other oral cavity sites; this practice obscures the distinctive biology, metastasis pattern, and treatment response of lip tumors (22, 23). By extracting 6,717 lip-only cases from SEER for 2010 to 2020, we created the first nationwide, modern-era benchmark that clinicians and researchers can use when counseling patients or designing trials.

Demographic modifiers have generally been acknowledged but not quantified for lip cancer; we showed that patients younger than 50 years old have an 87% lower hazard of death than those ages 75 or above and that White and Asian/Pacific Islander patients enjoy significant survival advantages over AI/AN patients. These disparities highlight inequities that merit targeted prevention and earlier detection strategies.

Fourth, the real-world effect of treatment choices has hitherto been described only in small institutional series (24–26); and our data link regional lymph node evaluation and surgical management with markedly improved outcomes, supporting guideline recommendations for elective neck treatment even when the primary tumor appears early.

Finally, we identified a pragmatic problem: more than 70% of SEER lip cases lacked complete T, N, or M information. By quantifying this deficit, we can provide registries and clinicians with an objective target for quality improvement.

### Demographic factors influencing survival

#### Gender disparity

A marked gender disparity emerged, with males exhibiting a consistently higher incidence than females across the decade (27, 28). Female patients achieved a marginal but statistically significant survival benefit, echoing broader head-and-neck literature that links hormonal, behavioral, and social factors to outcome. This is likely associated with increased occupational or recreational sun exposure among men (29). Racially, most patients were White, consistent with previous data linking fair skin to higher UV susceptibility (22, 28). Age-specific increases were noted in the older population, consistent with cumulative UV exposure (30). However, some younger cases occurred, pointing to changing lifestyle risk factors, including earlier tobacco or alcohol use or extended outdoor activities (29). Widowed status independently correlated with worse survival, emphasizing the influence of psychosocial support on adherence and follow-up. Routine psychosocial screening coupled with referral to support services should therefore accompany oncologic management.

#### Racial inequities

Table 4 confirms that AI or AN, Asian or Pacific Islander, and Black patients face adjusted hazards of death that are threefold, two-and-a-half–fold, and nearly threefold higher than those of White patients, respectively. These patterns mirror inequities documented for other head and neck sites and likely reflect intersecting barriers in access, comorbidity management, and timely treatment rather than inherent tumor biology (31, 32). Targeted outreach, culturally aligned navigation programs, and research addressing treatment utilization are warranted.

#### Age-specific considerations

In our study, the incidence of lip cancer increased with age, peaking in the sixth and seventh decades of life. Most cases were among participants ages 50 to 64 years and above. No deaths occurred in the three youngest age strata, precluding reliable hazard estimation but highlighting the excellent short-term prognosis of early-onset lip cancer. Longer follow-up and pooled data sets will be required to define late events in younger patients and explore whether distinctive molecular drivers underlie the disease in this age group. This trend may be due to the cumulative effects of carcinogenic exposure, such as UV radiation, over time and the biological aging process, which leads to DNA damage (33). Similarly, evidence from Brazilian populations shows that the majority of lip cancer cases are prevalent among individuals ages ≥60 years (33). Notably, we also observed an increase in cases among younger age groups, which is consistent with other studies that reported an increased incidence in middle-aged and younger patients (29, 34). Early exposure to risk factors, including sunlight during leisure activities such as fishing, tobacco smoking, and alcohol consumption at increasingly younger ages, may contribute to this trend. These findings emphasize the importance of early education and prevention strategies aimed at younger populations.

### Treatment modalities and survival outcomes

#### Lymph node metastasis and surgical intervention

Previous studies have shown that the rate of lymph node metastasis in patients with lip cancer ranges from 3% to 9% (32). In our study, we found that 70% of survivors and 30% of deaths had 1 to 3 nodes removed, whereas 65.5% of survivors and 34.4% of deaths had ≥4 nodes excised. The removal of more lymph nodes reflects efforts to control the spread of cancer, as lymph node metastasis is an indicator of disease progression and can significantly influence survival outcomes. The link between lymph node metastasis rates and extent of lymph node removal highlights the role of surgical intervention in both staging and managing more advanced cases of lip cancer.

#### Radiotherapy and chemotherapy

Among survivors, 67.3% received radiation after surgery compared with 32.6% of nonsurvivors, whereas very few patients underwent surgery both before and after radiation. This aligns with previous evidence, suggesting that surgical resection is the preferred treatment for early and small lesions. In contrast, larger tumors may require more extensive resection, which can affect the functional outcomes (35). Radiotherapy has disadvantages for early-stage lesions, such as prolonged treatment duration and potential complications, such as osteoradionecrosis of the mandible, which can limit future reconstruction options (36).

Our analysis showed that the majority of the survivors (72.2%) did not receive chemotherapy. Lip carcinoma is generally amenable to surgical resection, and surgery alone is often curative for early-stage primary lesions. Aggressive lesions such as T4 tumors typically require combined modality therapy with surgery and radiation, with the addition of chemotherapy in select cases (36). Our findings reflect the limited role of chemotherapy in treating lip cancer, reserved for advanced stage or recurrent disease.

### Influence of treatment sequencing

Exploratory comparisons indicated lower crude mortality among patients who underwent elective cervical lymph node sampling (one to three nodes removed) than among those who received more extensive dissections, consistent with guideline recommendations for early regional assessment. Conversely, individuals who received systemic therapy after surgery experienced a higher death proportion, almost certainly reflecting selection for aggressive or recurrent disease. The SEER database lacks regimen details, so prospective studies that capture indication, timing, and dose intensity are necessary before firm therapeutic conclusions can be drawn.

### Survival analysis by staging

The findings from this large SEER-based cohort confirm that clinical stage at diagnosis remains the strongest predictor of survival in patients with lip cancer. The striking disparity in outcomes—in which localized-stage patients experienced a 94% lower hazard of cancer-specific death (HR = 0.061) and regional-stage patients showed a 51% reduction (HR = 0.489) compared with those with distant disease—highlights the importance of early detection and diagnosis. These results are consistent with prior literature that highlights the prognostic primacy of tumor extent at presentation in head and neck cancers, including lip malignancies (11, 22).

The median survival time of 18 months for distant-stage patients aligns with global estimates, reflecting the aggressive course once metastatic spread occurs (14). In contrast, regional disease patients showed a median survival of 97 months, indicating the potential for long-term control with appropriate locoregional management. These findings reinforce the recommendations from the National Comprehensive Cancer Network Clinical Practice Guidelines and prior analyses, which advocate for early surgical excision and regional nodal assessment, particularly in T2 to T3 lesions, in which subclinical nodal spread is possible (13, 36).

High censoring rates, especially in the localized stage (92.4%), suggest prolonged survival durations beyond the observation period for most early-stage patients. This pattern further highlights the indolent behavior of many lip tumors, particularly those diagnosed at early stages, which are often well-differentiated and amenable to curative resection (12, 34).

The strong statistical significance of the Kaplan–Meier log-rank test (*χ*^2^ = 814.338, *P* < 0.001) and the robust hazard estimates from Cox regression (omnibus *χ*^2^ = 812.472, *P* < 0.001) validate the impact of stage on survival and support its continued inclusion as a central variable in both clinical and epidemiologic risk models (7, 10).

Our findings add to the growing body of evidence that aggregating lip cancer with other oral cavity malignancies masks unique prognostic features, including more favorable stage distribution at diagnosis and longer survival outcomes (2, 18). Given that more than 90% of cases were diagnosed at the localized stage, targeted public health measures, such as UV protection education and routine skin and lip examinations in high-risk populations (e.g., outdoor workers, fair-skinned elderly males, etc.), can further reduce the burden of advanced-stage presentations.

In line with our results, the limited incremental prognostic value of histologic grade once stage and race were adjusted further emphasizes the dominant role of staging in survival prediction. This finding aligns with large population-based studies, which have shown that once key clinical variables, such as TNM stage and nodal involvement, are accounted for, histologic grade does not significantly influence survival trajectories in lip or oral cavity SCC (37, 38).

### Survival analysis by histologic grading

#### Histologic grade offers limited incremental information

Although univariate comparisons detected survival differences among grade categories, the multivariable model in Table 4 indicates that grade does not independently influence outcome (all *P* > 0.05). This finding suggests that routine pathology reports can prioritize margin status and perineural invasion over histologic grade when counseling patients, provided that standardized grading remains available for research and quality assurance. This lack of independent prognostic value for histologic grade mirrors findings from two large population-based cohorts: a province-wide Alberta Cancer Registry study of 262 oral cavity SCCs diagnosed between 1998 and 2009, in which grade was excluded from their multivariable Cox model after stage, nodal status, and margin status absorbed its effect (37). The second study is an Australian series of 116 consecutively treated oral cancers reported in which grade likewise failed to enter the final model once stage and extracapsular spread were considered (38).

The analysis of survival outcomes by tumor grade in our cohort revealed that histologic differentiation significantly stratified patients in univariate Kaplan–Meier analysis but failed to retain statistical significance in multivariable Cox regression. Specifically, whereas grade III (poorly differentiated) tumors exhibited a notably lower proportion of censored (i.e., surviving) cases (63.5%) compared with grade I (92.2%), the corresponding HRs lacked statistical significance. These nonsignificant HRs, coupled with wide CIs, likely stem from limited events in higher-grade tumors and underline the challenge of deriving conclusive inferences in low-incidence subsets. This pattern mirrors earlier findings in population-level studies in which the prognostic contribution of histologic grade was diminished after adjusting for stage and race (39, 40). The nonsignificant adjusted HRs for grade II (HR = 0.532, *P* = 0.375) and grade III (HR = 1.555, *P* = 0.539) in our study suggest that tumor grade may act as a surrogate for stage or tumor biology rather than as an independent determinant of survival (41).

Moreover, the inability to estimate median survival times due to high censoring further limits clinical utility. These findings support the notion that whereas tumor grading remains essential for pathologic characterization, its role in prognostication may be subordinate to tumor stage and demographic modifiers (42). Accordingly, future studies should consider integrating molecular markers or genomic classifiers to better stratify risk, especially for high-grade or anaplastic variants in which conventional histopathologic grading may fall short.

### Survival analysis by race

Our analysis revealed pronounced racial disparities in lip cancer survival, underscoring the persistent influence of demographic factors on health outcomes. Among 5,305 patients analyzed, non-White individuals, specifically AI/AN, Asian or Pacific Islander, and Black populations, experienced significantly elevated risks of mortality compared with White patients.

The survival disadvantage was most severe among AI/AN patients, who demonstrated a threefold increased hazard of cancer-specific death (HR = 3.21; 95% CI, 1.43–7.18), followed by Black (HR = 2.70; 95% CI, 1.64–4.45) and Asian or Pacific Islander patients (HR = 2.45; 95% CI, 1.61–3.73). These disparities remained statistically significant even after adjusting for other prognostic variables, including stage and histologic grade, highlighting race as an independent predictor of survival.

Notably, the high proportion of censored cases among White patients (90.5%) and the comparatively lower proportion among Black patients (75.0%) may reflect differential follow-up durations or disparities in access to longitudinal care. Moreover, median survival could only be estimated for Black individuals (114 months), whereas it remained nonestimable for other racial groups due to high censoring. These findings indicate potential underestimation of survival disparities.

These findings align with broader evidence of racial inequities in head and neck cancer care, often attributed not to tumor biology but to systemic barriers such as delayed diagnosis, inadequate access to specialized care, insurance gaps, and socioeconomic disadvantages (24–26). Previous studies have demonstrated that minority populations are more likely to present at advanced stages and less likely to receive guideline-concordant therapy, contributing to poorer outcomes (25, 26).

Whereas the small sample size for AI/AN patients (*n* = 27) limits statistical precision, the elevated HR mirrors trends seen across SEER-based analyses of other head and neck malignancies (24, 43). Future studies should prioritize the inclusion of underrepresented populations and explore underlying causes, including geographic healthcare access, cultural barriers, and health literacy, to inform equity-focused interventions.

These findings collectively highlight the urgent need for targeted public health strategies aimed at early detection, culturally competent care delivery, and structural improvements in healthcare access to mitigate racial disparities in lip cancer outcomes (16, 26).

This study has several notable strengths that enhance the validity and relevance of its findings. First, the use of a large, population-based cohort comprising 6,717 patients with lip cancer from the SEER database (2010–2020) provides substantial statistical power and ensures generalizability to the broader US population.

A key strength of this study lies in the site-specific focus on lip cancer, in contrast to prior studies that grouped lip malignancies with other oral cavity cancers. By isolating lip cancers, this analysis offers a more accurate characterization of their unique demographic patterns and prognostic features.

This study has several limitations inherent to retrospective analyses using registry data. A significant proportion of TNM staging data was missing, with approximately 70% of cases lacking complete TNM codes. As a result, the analysis relied on the SEER Summary Stage, which, although validated, offered less granularity than the full TNM classification. The lack of detailed treatment data in SEER is another constraint. Information on surgical margins, radiation dose and field, chemotherapy agents, and systemic therapies is unavailable, limiting deeper insight into treatment effectiveness and mechanisms of failure.

Finally, the study was limited by the underrepresentation of certain racial and ethnic groups, such as AI/AN and Black patients. This may affect the precision of the disparity estimates. Moreover, racial misclassification, which is a recognized limitation in cancer registries, may further obscure the true extent of inequities in outcomes. Further research incorporating patient-level factors, genetic markers, and molecular profiles of lip cancer could improve prognosis (34).

The present data confirm that stage at diagnosis dominates prognosis, histologic grade adds little once stage and race are considered, and substantial racial and social disparities persist. Clinicians should prioritize early excision and elective neck evaluation, particularly in populations at risk of delayed presentation. Researchers should focus on strategies to improve TNM completeness, extend follow-up to younger patients, and elucidate modifiable factors that drive racial inequities. These efforts will refine personalized care pathways for this uncommon, though clinically significant, malignancy.

### Conclusion

This population-based analysis of 6,717 lip cancer cases from the 2010 to 2020 SEER cohort confirmed that the extent of disease at diagnosis remained the single most important prognostic factor. Localized lesions carried a 94% reduction in cause-specific mortality compared with distant metastasis (HR = 0.061; 95% CI, 0.044–0.086), and regional disease carried a 51% reduction (HR = 0.489; 95% CI, 0.339–0.707). Pronounced racial disparities persisted; AI/AN patients experienced more than a threefold hazard of death relative to White patients (HR = 3.21; 95% CI, 1.43–7.18), with elevated risks also evident for Asian or Pacific Islander (HR = 2.45) and Black patients (HR = 2.70). No deaths occurred in the three youngest age strata, preventing reliable estimation of age effects but highlighting the need for a longer follow-up- in younger patients.

Exploratory treatment comparisons indicated that elective sampling of one to three cervical lymph nodes coincided with a lower crude death proportion than extensive dissections, lending population-level support to guidelines that advocate early regional assessment of the neck. More than 70% of registry entries lacked at least one TNM component, which limited detailed staging analyses and highlights an urgent quality improvement target for cancer registries.

These findings collectively establish contemporary, lip-specific survival benchmarks that refine risk stratification by demonstrating the limited incremental value of histologic grade and quantify racial inequities that demand targeted early detection and treatment initiatives. Enhanced completeness of TNM reporting and routine capture of treatment variables would further strengthen future prognostic research and guide equitable care for this uncommon, although clinically significant, malignancy.
